# Forced expression of the DEK-NUP214 fusion protein promotes proliferation dependent on upregulation of mTOR

**DOI:** 10.1186/1471-2407-13-440

**Published:** 2013-09-27

**Authors:** Carl Sandén, Malin Ageberg, Jessica Petersson, Andreas Lennartsson, Urban Gullberg

**Affiliations:** 1 Department of Hematology, Lund University, BMC B13, Klinikgatan 26, 221 84 Lund, Sweden; 2 Center for Biosciences, Department of Biosciences and Medical Nutrition, Karolinska Institute, Novum 141 83, Huddinge, Sweden

**Keywords:** Acute myeloid leukemia, DEK-NUP214, DEK-CAN, Fusion gene, Proliferation, mTOR, Everolimus

## Abstract

**Background:**

The t(6;9)(p23;q34) chromosomal translocation is found in 1% of acute myeloid leukemia and encodes the fusion protein DEK-NUP214 (formerly DEK-CAN) with largely uncharacterized functions.

**Methods:**

We expressed DEK-NUP214 in the myeloid cell lines U937 and PL-21 and studied the effects on cellular functions.

**Results:**

In this study, we demonstrate that expression of DEK-NUP214 increases cellular proliferation. Western blot analysis revealed elevated levels of one of the key proteins regulating proliferation, the mechanistic target of rapamycin, mTOR. This conferred increased mTORC1 but not mTORC2 activity, as determined by the phosphorylation of their substrates, p70 S6 kinase and Akt. The functional importance of the mTOR upregulation was determined by assaying the downstream cellular processes; protein synthesis and glucose metabolism. A global translation assay revealed a substantial increase in the translation rate and a metabolic assay detected a shift from glycolysis to oxidative phosphorylation, as determined by a reduction in lactate production without a concomitant decrease in glucose consumption. Both these effects are in concordance with increased mTORC1 activity. Treatment with the mTORC1 inhibitor everolimus (RAD001) selectively reversed the DEK-NUP214-induced proliferation, demonstrating that the effect is mTOR-dependent.

**Conclusions:**

Our study shows that the DEK-NUP214 fusion gene increases proliferation by upregulation of mTOR, suggesting that patients with leukemias carrying DEK-NUP214 may benefit from treatment with mTOR inhibitors.

## Background

Acute myeloid leukemia (AML) is characterized by the dysregulated proliferation and impaired differentiation of myeloid precursor cells. Many of these leukemias harbor genetic translocations, which determine both the molecular mechanistics and the prognosis of the disease [1]. The t(6;9)(p23;q34) chromosomal translocation is found in 1% of AML, where it is associated with young age and poor prognosis [2]. The translocation occurs between specific introns in the gene *DEK* on chromosome 6 and the gene *NUP214* on chromosome 9, creating the fusion gene *DEK-NUP214* (formerly *DEK-CAN*). The original reading frames are preserved, yielding an invariable fusion protein comprising almost the entire chromatin remodeling protein DEK and the carboxy-terminal two thirds of the nucleoporin NUP214 [3].

Despite extensive characterization of many other fusion genes, the role of DEK-NUP214 is still poorly understood. We have previously shown that DEK-NUP214 promotes the activating phosphorylation of the eukaryotic translation initiation factor 4E (eIF4E) on Ser^209^ and increases the protein synthesis of myeloid cells [4]. However, the cause as well as the cellular effects thereof remain to be explored. Recently, DEK-NUP214 has been shown to induce leukemia in a murine model, but only from long-term repopulating stem cells and with long latency, emphasizing the need for cooperating mutations [5]. A striking feature of leukemias with the *DEK-NUP214* fusion gene is the concomitant internal tandem duplication (ITD) in the tyrosine kinase FLT3. The *FLT3-ITD* genotype is more than three times as common in leukemias with t(6;9)(p23;q34) as in other AML [2,6]. This suggests a classic oncogenic cooperation between a pro-proliferative FLT3-ITD and a differentiation-blocking DEK-NUP214. However, recent studies have identified a role for FLT3-ITD also in inhibition of myeloid differentiation [7]. And contrary to many fusion proteins observed in AML, DEK-NUP214 does not seem to inhibit differentiation, at least not when expressed in the monocytic cell line U937 [8]. This raises the possibility of a role for DEK-NUP214 in proliferation.

The mechanistic target of rapamycin (mTOR) is a central node in the regulation of both proliferation and translation [9]. The mTOR protein is found in two complexes. Activated by growth factor signaling, the mTOR complex 2 (mTORC2) phosphorylates Akt at Thr^450^ and Ser^473^, in turn activating mTOR complex 1 (mTORC1) [10]. mTORC1 initiates translation by phosphorylation of its downstream targets, such as the p70 S6 kinase [11]. Although mTORC1 regulates the translation of most mRNAs, some transcripts are particularly sensitive. These include many oncogenes such as c-myc and cyclin D1. Activation of the mTORC1 pathway thus promotes cellular growth and proliferation [12,13].

In addition to its role in translation, mTORC1 also affects cellular metabolism by promoting the more energy-efficient oxidative phosphorylation over glycolysis. This role is independent of the translational regulation machinery and rather seems to involve phosphorylation of mitochondrial enzymes [14,15]. Due to its multiple roles in carcinogenesis and its common overactivation in cancer, mTOR has become an attractive target for cancer therapy and there are currently several inhibitors in clinical trials [16]. Recently, the FDA approved the highly specific mTORC1 inhibitors everolimus (RAD001) and temserolimus (CCI-779) for the treatment of advanced renal cell carcinoma and everolimus is currently in clinical trial for acute myeloid leukemia [17-19].

In this study, we show that overexpression of DEK-NUP214 in the myeloid cell line U937 leads to increased levels of mTOR and activation of the mTOR target p70S6K. This translates into higher protein synthesis and a metabolic shift from glycolysis to oxidative phosphorylation. Accordingly, cells expressing DEK-NUP214 proliferate faster than their normal counterparts. Treatment with the mTORC1 inhibitor everolimus selectively reverses the DEK-NUP214-induced proliferation, suggesting that the effect is mTOR-dependent and that patients with t(6;9)(p23;q34) may be suitable for treatment with mTOR inhibitors.

## Methods

### Cell culture

The cell lines U937 and PL-21 (ATCC, Manassas, VA, USA) and stable clones derived thereof were cultured in RPMI 1640 medium (Life Technologies, Carlsbad, CA, USA) supplemented with 10% fetal bovine serum (Life Technologies). Stable clones expressing either the DEK-NUP214 fusion gene [4], DEK-NUP214 deletion mutants [4] or the corresponding empty pcDNA3 vector, were generated by electroporation followed by incubation for 48 h and subsequent seeding of 10 000 cells per well in 100 μl medium. After two weeks of selection by culture in growth medium supplemented with 0.5 mg/ml geneticin (Life Technologies), clones were selected and expanded.

### Proliferation experiments

For proliferation experiments, cells were seeded in fresh culture medium at a density of 0.5 × 10^6^ cells/ml and when indicated treated with daily additions of the mTORC1 inhibitor everolimus (Sigma-Aldrich, St. Louis, MO, USA). Cell counting was performed with the Countess Automated Cell Counter (Life Technologies) and viability was determined on the basis of trypan blue dye exclusion (Life Technologies).

### Protein expression

Protein expression was analyzed by western blot one day after seeding, as described above. Cells were washed in PBS (PAA Laboratories, Pasching, Austria), resuspended and frozen in sample buffer containing 0.1 M Tris–HCl pH 6.8, 0.2 M β-mercaptoethanol, 14% glycerol (v/v), 3% SDS (w/v), 0.01% bromophenol blue (w/v), Complete protease inhibitor cocktail (Roche Diagnostics GmbH, Mannheim, Germany) and PhosStop protease inhibitor cocktail (Roche Diagnostics GmbH). Samples were sonicated in a UP50H ultrasonic homogenizer (Dr. Hielscher GmbH, Teltow, Germany), boiled for 5 minutes and centrifuged at 14 000 × g for 5 minutes. Lysates corresponding to 500 000 cells were run on tris-glycine gels (Life Technologies) and transferred by an SV20-SDB semi-dry blotter (Sigma-Aldrich) to Hybond ECL membrane (GE Healthcare, Uppsala, Sweden). Membranes were blocked with 5% bovine serum albumin (Sigma-Aldrich) and incubated with one of the following antibodies according to the manufacturers’ recommendations: anti-α-tubulin (Sigma-Aldrich), anti-GAPDH (Santa Cruz Biotechnology, Santa Cruz, CA, USA), anti-phospho-mTOR-Ser^2448^, anti-mTOR, anti-phospho-Akt-Ser^473^, anti-phospho-Akt-Thr^308^ or anti-phospho-p70-S6K-Thr^389^ (Cell Signaling Technology, Danvers, MA, USA). HRP-conjugated anti-mouse or anti-rabbit were used as secondary antibodies (Bio-Rad Laboratories, Hercules, CA, USA) and detected with the EZ-ECL kit (Biological Industries, Kibbutz Beit Haemek, Israel). Quantification was performed using the Molecular Imager FX (Bio-Rad Laboratories) with the Quantity One 4.2.2 software (Bio-Rad Laboratories).

### Gene expression analysis

Gene expression was determined by quantitative real-time PCR. RNA was extracted using the RNeasy Mini Kit (Qiagen GmbH, Hilden, Germany) and reverse transcription was performed with the High Capacity cDNA Reverse Transcription Kit (Life Technologies). Expression levels were assayed with the TaqMan Gene Expression Assay and primer-probe pairs for the detection of glyceraldehyde-3-phosphate dehydrogenase (GAPDH; Hs99999905_m1), mechanistic target of rapamycin (mTOR; Hs00234522_m1) or DEK-NUP214 (custom made) (Life Technologies). The amplification reaction was performed using the StepOnePlus Real-Time PCR System (Life Technologies). The expression of DEK-NUP214 and mTOR was calculated relative to the expression of GAPDH using the comparative C_T_ method, as previously described [20]. cDNA from a patient with the t(6;9)(p23;q34) chromosomal translocation was kindly provided by professor Bertil Johansson at the Department of Clinical Genetics, Lund University.

### Global translation assay

The translation rates of the stable clones were assessed by radioactive labeling of newly synthesized proteins. Cells were seeded in fresh culture medium at a density of 0.5 × 10^6^ cells/ml. At indicated time points, EXPRESS^35^S Protein Labeling Mix containing radioactively labeled methionine and cysteine (PerkinElmer, Waltham, MA, USA), was added to cell cultures to a final concentration of 50 μCi/ml. After incubation for 2 h, 100 000 viable cells of each clone were sorted by a FACSAria cell sorter (BD Bioscience, San José, CA, USA), washed in PBS and lysed in radioimmunoprecipitation buffer (30 nM HEPES, pH 7.3, 1% Triton-X (v/v), 1% sodium deoxycholate (w/v), 0.1% SDS (w/v), 0.15 M NaCl) containing the Complete Protease Inhibitor Cocktail (Roche Diagnostics GmbH, Mannheim, Germany). Proteins were precipitated by addition of trichloroacetic acid to a final concentration of 9%. The precipitate was washed twice in acetone, suspended in 50 μl 0.1 M Tris–HCl, pH 8.6, and added to 5 ml of scintillation fluid (Beckman Coulter, Fullerton, CA, USA). The radioactivity of the samples was measured by a Wallac Guardian 1414 liquid scintillation counter (PerkinElmer). Values were corrected for background by subtracting the values from samples incubated with the EXPRESS^35^S Protein Labeling Mix on ice.

### Metabolic assays

Cells were seeded in fresh culture medium at a density of 0.5 × 10^6^ cells/ml. At indicated time points, cell suspension was taken out and centrifuged at 145 × g for 5 minutes. Supernatant was collected and stored at −80°C to prevent degradation of lactate. The glucose concentration was measured by applying 10 μl of supernatant to the Glucose Assay Kit II (BioVision, Mountain View, CA, USA). After dilution of the supernatant 1:50 in lactate assay buffer, the lactate concentration was determined by applying 10 μl to the Lactate Assay Kit II (BioVision). Absorbance was measured at 450 nm with a Labsystems Multiskan Plus Plate Reader (Thermo Fisher Scientific, Waltham, MA, USA).

### Statistical analysis

Statistical testing was performed using the two-tailed t test, where the averages of the three DEK-NUP214 clones from each experiment were tested against the averages of the three control clones from the same experiments. Stars represent conventional significance levels; single stars indicate p < 0.05, double stars indicate p < 0.01 and triple stars indicate p < 0.001.

## Results

### Stable expression of DEK-NUP214 in myeloid cell lines

**Figure 1:**
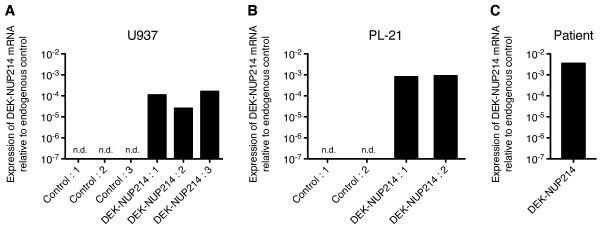
**Expression of the DEK-NUP214 fusion gene.** Expression of DEK-NUP214 mRNA by quantitative real-time PCR in the generated stable clones of **(A)** U937 and **(B)** PL-21, as well as **(C)** a patient sample expressing the fusion gene. Values were calculated relative to the expression of the endogenous control, which did not differ between the clones. N.d. denotes not detected.

To investigate the influence of DEK-NUP214 on cellular functions, we expressed the fusion gene in the myeloid cell lines U937 and PL-21 and generated stable clones. Expression of *DEK-NUP214* was verified by real-time PCR (Figure 1A-B). To ensure that the overexpression was in the same range as endogenously expressed DEK-NUP214, we quantified the expression of *DEK-NUP214* in a sample from a patient with the t(6;9)(p23;q34) (Figure 1C). The expression levels in the cell lines were on average 11% of that in the patient sample and all clones expressed less DEK-NUP214 than the patient sample, thus reducing the risk of overexpression artifacts. Protein expression from the DEK-NUP214 vector was verified by in vitro translation (data not shown). As previously reported, the fusion protein does not allow detection by western blot, using antibodies against either DEK or NUP214 [5]. However, the verified expression of DEK-NUP214 mRNA by real-time PCR together with the expression of the geneticin resistance protein from the same vector during clonal selection supports that the fusion protein is expressed in the stable clones.

### DEK-NUP214 stimulates the proliferation of U937 and PL-21

**Figure 2:**
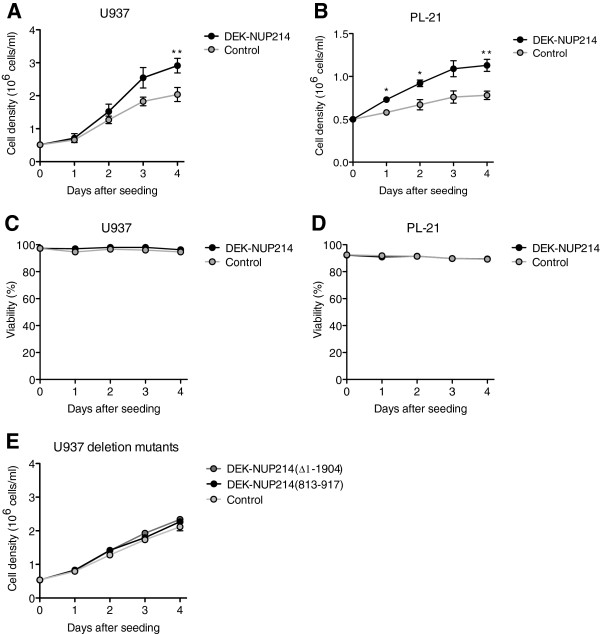
**DEK-NUP214 promotes proliferation.** Proliferation of **(A)** U937 and **(B)** PL-21 cells. Viability of the **(C)** U937 and **(D)** PL-21 cell cultures, as determined by trypan blue dye exclusion. **(E)** Proliferation of U937 cells expressing deletion mutants of DEK-NUP214. Mean values were calculated from three independent experiments, containing all clones. Error bars represent S.E.M.

To evaluate the leukemia-associated properties of DEK-NUP214, we first studied its effect on proliferation. Stable clones constitutively expressing either *DEK-NUP214* or the empty vector were seeded in fresh culture medium and followed for four days with regard to cell number and viability. The results show that both U937 and PL-21 cells expressing DEK-NUP214 expand faster than the respective control cells (Figure 2A and B). Since the viability did not differ between the DEK-NUP214 and the control clones (Figure 2C and D), the difference in cell density was not the result of decreased cell death but rather that of increased proliferation. As the DEK-NUP214 and control cells did not differ in cell cycle distribution (data not shown), the increased proliferation can be attributed to a symmetrical decrease of the major cell cycle phases rather than the shortening of one particular phase. To determine whether the proliferative effect of DEK-NUP214 is dependent on the entire fusion gene, we performed the proliferation experiment with previously established deletion mutants of DEK-NUP214 in U937 cells [4]. To assess the contribution of the *NUP214* part of the fusion, we used two constructs containing *DEK* fused with only either the N-terminal dimerization domain of *NUP214*, DEK-NUP214(813–917), or the C-terminal CRM1-binding domain of *NUP214*, DEK-NUP214(∆1-1904). Both of these constructs failed to reproduce the proliferative effect of the complete fusion (Figure 2E), demonstrating the importance of *NUP214* for the proliferative effect of the fusion gene. However, overexpression of *NUP214* alone has been shown to induce growth arrest and apoptosis in U937 cells [8], demonstrating that also the *DEK* part of the fusion is essential for the proliferative effect of the *DEK-NUP214* fusion gene.

### DEK-NUP214 promotes mTOR signaling

**Figure 3:**
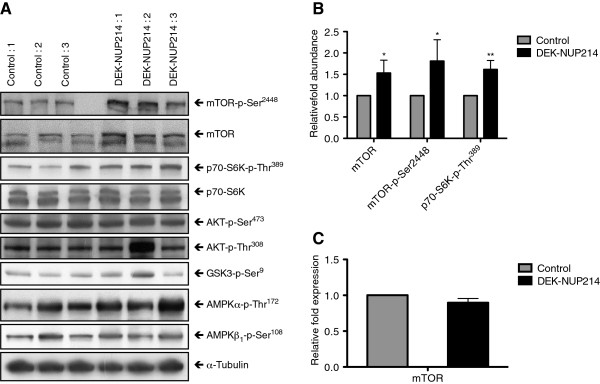
**DEK-NUP214 increases the expression of mTOR. A**. Phosphorylated and total protein levels for components of the mTOR signaling pathway in U937 cells one day after seeding, showing increased levels of mTOR and activation of the mTORC1 substrate p70S6K without activation of the mTORC2 substrate Akt. α-tubulin is used as an equal loading control. Images show one representative experiment out of three. **B**. Protein abundance levels of cells expressing DEK-NUP214, relative to control cells. The mean of three experiments, error bars represent S.E.M. **C**. Expression of the *mTOR* gene in DEK-NUP214 cells relative to control cells. The mean of three experiments, error bars represent S.E.M.

Cellular proliferation is regulated by a wide range of signaling pathways. But the effect on proliferation seen here and the effect on protein synthesis previously observed [4], suggested that DEK-NUP214 may act on the major regulator of translation, the mechanistic target of rapamycin, mTOR [21]. Analysis of the mTOR pathway revealed that cells expressing DEK-NUP214 have higher levels of both total mTOR protein and mTOR protein phosphorylated at Ser^2448^ (Figure 3A-C). To determine the impact of the increased mTOR levels on the activity of the two mTOR complexes, we analyzed the phosphorylation status of their downstream targets. The p70 S6 kinase is a substrate for mTORC1, activated by phosphorylation by mTOR at Thr^389^[22]. Concurrent with mTORC1 activation, we observed an increase in the level of phosphorylated p70S6K protein (Figure 3A and B). However, the mTORC2-mediated phosphorylation of Akt at Ser^473^ was not affected by the expression of DEK-NUP214 (Figure 3A), suggesting that the increased levels of mTOR in this case primarily leads to increased mTORC1 activity. To characterize the mTOR increase, we analyzed the transcription of the *mTOR* gene by real-time PCR. This was unaffected by DEK-NUP214 (Figure 3C), demonstrating that the increase in mTOR expression occurs on the post-transcriptional level. We proceeded to examine the upstream regulators of mTORC1. However, these did not display altered activation as determined by western blot against phosphorylated AMPK and GSK3 as well as the two activating phosphorylations of Akt at Thr^308^ and Ser^473^ (Figure 3A). The increased phosphorylation of Akt at Thr^308^ in the second DEK-NUP214 clone was not seen in the other DEK-NUP214 clones and was thus attributed to mechanisms other than the expression of the fusion protein. In conclusion, our protein expression data suggests that expression of the DEK-NUP214 fusion gene leads to higher protein levels of mTOR and increased signaling through the mTORC1 pathway.

### DEK-NUP214 increases protein synthesis

**Figure 4:**
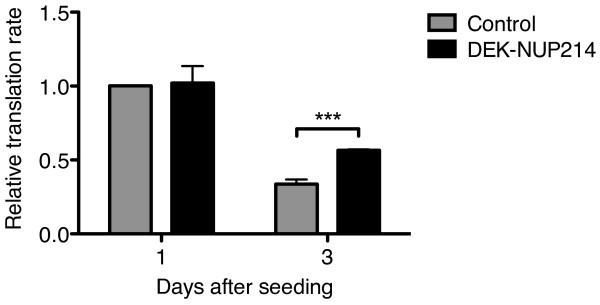
**DEK-NUP214 promotes translation.** Global protein synthesis as assayed by the incorporation of radioactively labeled amino acids into newly synthesized protein in U937 cells. All values are normalized to the level of the control cells one day after seeding. Bars represent the mean values of three experiments. Error bars represent S.E.M.

To determine the functional importance of the increased mTOR signaling, we proceeded to study protein translation. We performed a global translation assay where the incorporation of radioactively labeled amino acids into newly synthesized proteins reflects the rate of translation. The results show that one day after seeding in fresh culture medium, U937 cells expressing DEK-NUP214 and control cells both display high translation rates (Figure 4). However, three days after seeding, the translation rates have declined from their maxima and the effect of DEK-NUP214 becomes clear. The cells expressing DEK-NUP214 then had a 68% higher protein synthesis than the control cells (Figure 4). This demonstrates that DEK-NUP214 sustains a higher translation rate, in concordance with increased pro-translational signaling.

### DEK-NUP214 induces a metabolic shift

**Figure 5:**
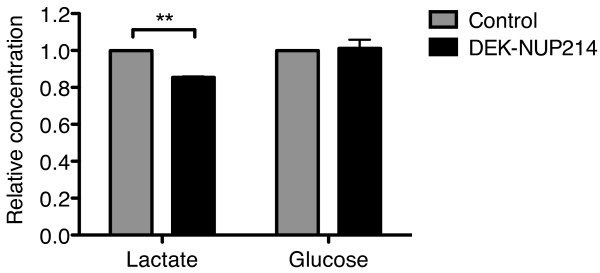
**DEK-NUP214 alters the balance between glycolysis and oxidative phosphorylation.** Relative concentrations of accumulated lactate and remaining glucose in cell supernatant three days after seeding, showing decreased glycolysis but sustained glucose consumption, indicating increased oxidative phosphorylation in U937 cells expressing DEK-NUP214. Bars represent mean values from three experiments, normalized to control cells. Error bars represent S.E.M.

In addition to its role in protein translation, mTOR also regulates glucose metabolism. In the balance between aerobic and anaerobic catabolism, mTORC1 promotes the more energy-efficient oxidative phosphorylation over glycolysis [15]. We therefore studied the metabolic properties of the cells by measuring the levels of lactate and glucose in the cell supernatant after three days of culture. The U937 cultures expressing DEK-NUP214 produced 15% less lactate, the by-product of glycolysis (Figure 5). However, the glucose consumption in these cultures was not proportionally reduced (Figure 5). Thus, this was not due to an overall reduction in glucose metabolism. Rather, it suggests a shift in the balance from glycolysis to oxidative phosphorylation, consistent with increased mTORC1 signaling.

### The proliferative effect of DEK-NUP214 is dependent on mTOR

**Figure 6:**
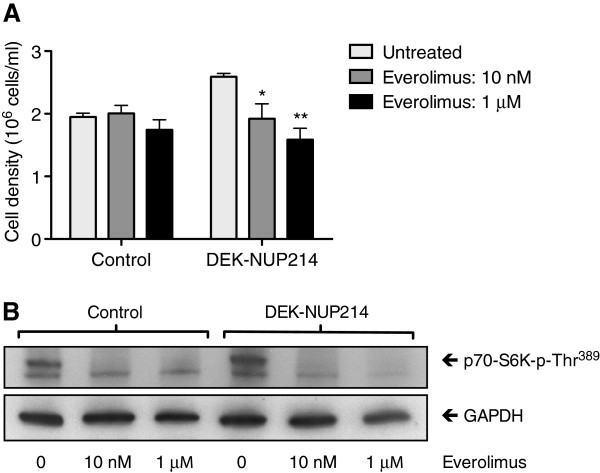
**The proliferative effect of DEK-NUP214 depends on mTOR. A**. Proliferation as determined by U937 cell density three days after seeding and with daily additions of the mTORC1 inhibitor everolimus. Viability was unaffected by the treatment. Bars represent mean values from four experiments. Error bars represent S.E.M. **B**. Potency of the mTORC1 inhibitor everolimus, as determined by reduced phosphorylation of the direct mTORC1 target p70 S6 kinase.

Given the emergence of mTOR inhibitors with clinical potential, we proceeded to determine the importance of the upregulated mTOR for the proliferative effect of DEK-NUP214. Treatment of the U937 cells with the mTORC1 inhibitor everolimus strikingly ablated the proliferation increase by DEK-NUP214. When treated with daily doses of everolimus, the proliferation of cells expressing DEK-NUP214 was reduced to the level of the control cells, whose proliferation was unaffected (Figure 6A). The effect of everolimus was anti-proliferative rather than pro-apoptotic, as the viability was consistently above 90%, similar between DEK-NUP214 and control cells, and not reduced by the treatment (data not shown). In the higher dose of 10 μM, everolimus was toxic to both DEK-NUP214 and control cells, proving its efficacy also against leukemia cells not expressing this fusion protein (data not shown). The potency of the mTORC1 inhibitor was determined by western blot, where treatment with everolimus markedly reduced the level of phosphorylated p70 S6 kinase (Figure 6B). Everolimus also ablated the difference in p70S6K phosphorylation between DEK-NUP214 and control cells, analogous to its effect on proliferation.

## Discussion

This study is the first to demonstrate that the expression of the fusion gene DEK-NUP214 leads to increased cellular proliferation. We show that this is dependent on upregulation of the signal transduction protein mTOR with subsequent effects on protein synthesis and glucose metabolism. We proceed to show that the proliferative effect can be overcome by inhibition of mTORC1 with everolimus, suggesting that patients with the *DEK-NUP214* fusion gene may benefit from treatment with mTOR inhibitors.

The biology of DEK-NUP214 is notoriously elusive. Although the genetic translocation was characterized almost two decades ago, only a few reports have studied its role in leukemogenesis and none has been able to show whether the contribution is on the level of proliferation or differentiation. We find in this study that DEK-NUP214 increases the proliferation of myeloid cells. This is a property shared by several fusion proteins, the most similar being SET-NUP214, which contains the same portion of NUP214 [23]. But also other nucleoporin fusions such as NUP98-HOXA9 and NUP98-HHEX show similar pro-proliferative properties both in culture and in vivo [24-26]. In some aspects, this finding is in contrast with a previous study of the NUP214 gene, which also included one DEK-NUP214 clone. This clone displayed equal or slightly lower proliferation as compared to wild-type cells [8]. We cannot with certainty determine the reason for this discrepancy but it may be the result of different expression levels of the fusion gene. Interestingly, Boer et al. selected the clone with the highest inducible expression of DEK-NUP214 for their proliferation experiment. As with some other oncogenes, DEK-NUP214 may promote proliferation at low or moderate levels and inhibit proliferation when highly expressed. Such a disadvantageous effect of high gene expression could also explain the low expression levels of DEK-NUP214 in cells with stable expression of the gene; both our clones and cells from patients with the t(6;9)(p23;q34) translocation [27].

In characterizing the proliferative effect, we find that DEK-NUP214 promotes signaling through the mTOR pathway. We demonstrate that DEK-NUP214 increases the level of mTOR protein, without affecting any of the upstream regulators AMPK, GSK3 or Akt. Despite extensive characterization of mTOR activation, surprisingly little is known about the regulation of its expression. β-catenin is known to influence the transcription of mTOR [28] but since this was unaffected by DEK-NUP214, we suggest another mode of regulation. The mechanism remains to be investigated and may involve miRNA-mediated inhibition of translation, subcellular relocalization or covalent modification, but most likely involves the stabilization of mTOR by protein-protein interaction, since this has been described for several other proteins such as Raptor [29], C/EBPδ [30], Tti1 [31] and Tel2 [31]. We also see an increase in the level of mTOR protein phosphorylated on Ser^2448^. This phosphorylation is mediated by p70S6K in a feedback loop, whose effect on the activity of mTOR is not yet understood [32,33]. The increase in mTOR-p-Ser^2448^ may arise from the observed activation of p70S6K or may reflect the increased availability of mTOR protein in cells expressing DEK-NUP214. By examining the phosphorylation of their substrates, we can conclude that in this context, the increased level of mTOR confers increased activity of mTORC1 but not mTORC2. The reason for this may be that the availability of the other factors of the complexes makes mTORC1 more susceptible to an mTOR increase or that the mTORC1 substrates are more sensitive to changes in mTOR complex activity.

To address the functional relevance of the increased mTOR signaling, we analyzed the cellular translation rate. The first day after seeding, nutrients and growth factors are readily available and the conditions for translation are highly favorable. The rate of translation is subsequently very high. Hence, it is not very surprising that the expression of DEK-NUP214 does not markedly enhance the translation rate at this time point. However, three days after seeding, the control cells have decreased their rate of protein synthesis by two thirds whereas the cells expressing DEK-NUP214 sustain a 68% higher translation rate than the control cells. Due to the rapid growth and proliferation of cancer cells, they require extensive protein synthesis also when nutrients and growth factors are scarce [34]. This key feature is crucial for malignant transformation and could be a mechanism by which DEK-NUP214 contributes to leukemogenesis.

A more recently discovered function of mTOR is in glucose metabolism. Most cancer cells initially rely heavily on aerobic glycolysis, a phenomenon known as the Warburg effect [35]. However, as proliferation increases, so does the energy demand. A second metabolic shift can serve to reestablish the more energy-efficient oxidative phosphorylation, while also providing metabolites for macromolecule anabolism [36]. Dysregulation of the mTOR pathway has been proposed as such an event, as overactivation of mTORC1 leads to a shift from glycolysis to oxidative phosphorylation [15]. Our findings confirm this notion. Cultures of cells expressing DEK-NUP214 produce less lactate but consume as much glucose as cultures of control cells, indicating such a shift. Given the higher proliferation rate and thus higher number of cells in the DEK-NUP214 cultures, the glucose consumption per cell is somewhat lower than for the control cells. However, this decrease alone cannot explain the decrease in lactate levels, since a reduction in glucose consumption that only offsets the effect of increased proliferation on total glucose levels would consequently also only offset the effect of proliferation on total lactate levels. What we observe here is a reduction in total lactate levels, thus indicating a metabolic shift from glycolysis to oxidative phosphorylation.

mTOR has attracted widespread attention as a target for cancer therapy and several variants of the original mTOR inhibitor rapamycin are being evaluated in clinical trials, both for solid tumors and leukemias [18]. One of these is everolimus, which employs the same mechanism of action as rapamycin and has been approved by the FDA for the treatment of renal cell carcinoma [17]. Our results show that treatment with everolimus completely ablates the proliferative phenotype induced by DEK-NUP214. Strikingly, it does so at concentrations that do not affect the control cells. This may be because the higher proliferation rate of the DEK-NUP214 cells produces higher demands and thus increased dependence on mTORC1 signaling. Compensatory pathways may thus be able to sustain the proliferation rate of the control cells but not the increase caused by the expression of DEK-NUP214. These results demonstrate that the increased proliferation by DEK-NUP214 is indeed dependent on mTORC1. Furthermore, it suggests that patients with leukemias harboring the t(6;9)(p23;q34) may benefit from treatment with the novel mTOR inhibitors that are becoming increasingly available.

## Conclusions

The DEK-NUP214 fusion gene is associated with poor prognosis in acute myeloid leukemia but its contribution to the disease remains largely unknown. In this study, we expressed DEK-NUP214 in the AML cell line U937 and show that this leads to increased expression of mTOR as well as increased phosphorylation of the mTORC1 substrate p70S6K but not the mTORC2 substrate Akt. Consistent with increased mTORC1 activation, the cells also display increased protein translation and a metabolic shift from glycolysis to oxidative phosphorylation. Cells expressing DEK-NUP214 also proliferate faster, a difference that is abrogated by treatment with the mTORC1 inhibitor everolimus at a concentration that does not affect the proliferation or the viability of the control cells. This demonstrates that the proliferative effect is dependent on mTOR and suggests that cells carrying the DEK-NUP214 fusion gene may be sensitive to treatment with the mTOR inhibitors currently being evaluated for the treatment of leukemia.

## Competing interests

The authors declare that they have no competing interests.

## Authors’ contributions

CS, MA, AL and UG designed experiments. CS, JP and AL performed experiments. CS, MA, JP, AL and UG analyzed data. CS and UG wrote the paper. All authors read and approved the manuscript.

## Pre-publication history

The pre-publication history for this paper can be accessed here:

http://www.biomedcentral.com/1471-2407/13/440/prepub
